# The GOLPH3 pathway regulates Golgi shape and function and is activated by DNA damage

**DOI:** 10.3389/fnins.2015.00362

**Published:** 2015-10-07

**Authors:** Matthew D. Buschman, Mengke Xing, Seth J. Field

**Affiliations:** Division of Endocrinology and Metabolism, Department of Medicine, University of CaliforniaSan Diego, La Jolla, CA, USA

**Keywords:** Golgi, phosphatidylinositol-4-phosphate, GOLPH3, MYO18A, secretory trafficking, DNA damage, cancer, neurodegenerative disease

## Abstract

The Golgi protein GOLPH3 binds to PtdIns(4)P and MYO18A, linking the Golgi to the actin cytoskeleton. The GOLPH3 pathway is essential for vesicular trafficking from the Golgi to the plasma membrane. A side effect of GOLPH3-dependent trafficking is to generate the extended ribbon shape of the Golgi. Perturbation of the pathway results in changes to both Golgi morphology and secretion, with functional consequences for the cell. The cellular response to DNA damage provides an example of GOLPH3-mediated regulation of the Golgi. Upon DNA damage, DNA-PK phosphorylation of GOLPH3 increases binding to MYO18A, activating the GOLPH3 pathway, which consequently results in Golgi fragmentation, reduced trafficking, and enhanced cell survival. The PtdIns(4)P/GOLPH3/MYO18A/F-actin pathway provides new insight into the relationship between Golgi morphology and function, and their regulation.

## Introduction

In many mammalian cell types the Golgi complex appears by light microscopy as an extended ribbon that wraps partially around the nucleus. The ribbon is composed of many subresolution stacks of membrane (as observed by electron microscopy) that are linked via Golgi-associated proteins. From first principles we can conclude that the steady-state appearance of the Golgi reflects the balance of forces applied to it. Changes in the shape of the Golgi presumably reflect alterations in the balance of forces applied to the Golgi. Since at least some of the forces that are applied to the Golgi are likely to be important for its function in vesicle trafficking, changes in trafficking machinery might be expected to lead to changes in Golgi morphology. However, it is important to bear in mind that the morphology of the Golgi varies significantly across species (reviewed in Mowbrey and Dacks, 2009; Wei and Seemann, 2010), suggesting that diverse morphologies can still be fully competent for trafficking.

**Figure 1:**
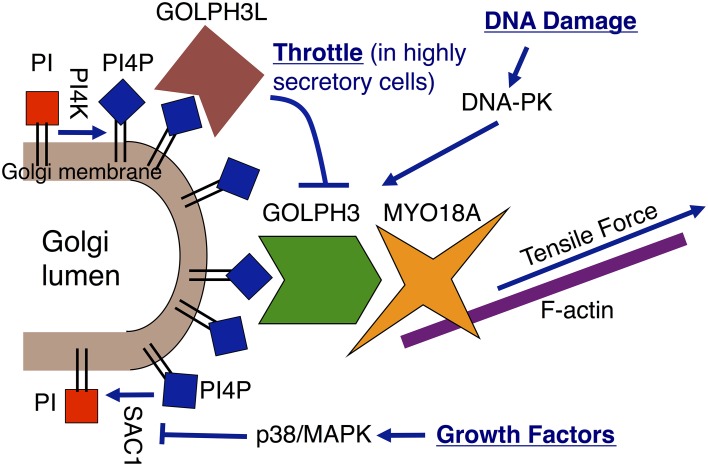
**Regulation of the Golgi via the GOLPH3 pathway**. The GOLPH3 pathway links the Golgi to the actin cytoskeleton, which applies a tensile force to the Golgi that is essential for anterograde trafficking. The GOLPH3 pathway is subject to regulation by different mechanisms. Growth factor signaling increases Golgi PtdIns(4)P levels through translocation of SAC1 away from the Golgi to the ER (Blagoveshchenskaya et al., 2008). GOLPH3L is a GOLPH3 paralog that acts as a dominant negative inhibitor of the GOLPH3 pathway due to its ability to bind to PtdIns(4)P, while being unable to bind to MYO18A (Ng et al., 2013). GOLPH3L acts as a throttle to Golgi-to-plasma membrane trafficking in highly secretory cells. Upon DNA damage, DNA-PK activates the pathway by phosphorylation of GOLPH3 to enhance its interaction with MYO18A (Farber-Katz et al., 2014).

The GOLPH3 pathway provides a link from the trans-Golgi membrane to the actin cytoskeleton that plays a critical role in anterograde trafficking to the plasma membrane (Figure 1). The trans-Golgi is highly enriched in phosphatidylinositol-4-phosphate (PtdIns(4)P) (Godi et al., 1999, 2004). In mammalian cells Golgi PtdIns(4)P is produced by the Golgi localized PI-4-kinases, PI-4-kinase-IIIβ (PI4KIIIβ) and PI-4-kinase-IIα (PI4KIIα) (Wong et al., 1997; Wang et al., 2003; De Matteis et al., 2005). PtdIns(4)P levels are reduced by the action of the Golgi/ER localized PtdIns(4)P-4-phosphatase, SAC1 (Foti et al., 2001; Schorr et al., 2001). GOLPH3 localizes to the trans-Golgi via its direct interaction with PtdIns(4)P (Dippold et al., 2009). This interaction is mediated by a binding pocket on the hydrophobic face of GOLPH3. Furthermore, GOLPH3's interaction with PtdIns(4)P and its Golgi localization are conserved from yeast to humans (Dippold et al., 2009; Wood et al., 2009). GOLPH3 also binds tightly and specifically to the unconventional myosin, Myosin 18A (MYO18A) (Dippold et al., 2009; Ng et al., 2013; Taft et al., 2013; Farber-Katz et al., 2014), and MYO18A has been shown to bind to F-actin (Isogawa et al., 2005; Guzik-Lendrum et al., 2013; Taft et al., 2013). Thus, GOLPH3/MYO18A serves to link the PtdIns(4)P-rich trans-Golgi membrane to the actin cytoskeleton.

## Perturbations of the GOLPH3 pathway alter trafficking and golgi morphology

All of the components of the pathway, PtdIns(4)P, GOLPH3, MYO18A, and F-actin, are required for efficient Golgi-to-plasma membrane trafficking. PtdIns(4)P has been shown to be required for Golgi secretory function across species from yeast to humans (Hama et al., 1999; Walch-Solimena and Novick, 1999; Audhya et al., 2000; Wang et al., 2003). Likewise, GOLPH3 and MYO18A are required for Golgi-to-plasma membrane trafficking as measured by VSVG delivery to the plasma membrane (Dippold et al., 2009), total secretory flux by pulse-chase analysis (Ng et al., 2013), secretion of hepatitis C viral particles (Bishé et al., 2012), and for exit of anterograde trafficking vesicles from the Golgi (Dippold et al., 2009). Finally, F-actin has been demonstrated to be required for Golgi secretory function (Hirschberg et al., 1998; Lázaro-Diéguez et al., 2007). Taken together, the data indicate that the majority of trafficking from the Golgi to the plasma membrane depends on the PtdIns(4)P/GOLPH3/MYO18A/F-actin pathway.

**Figure 2:**
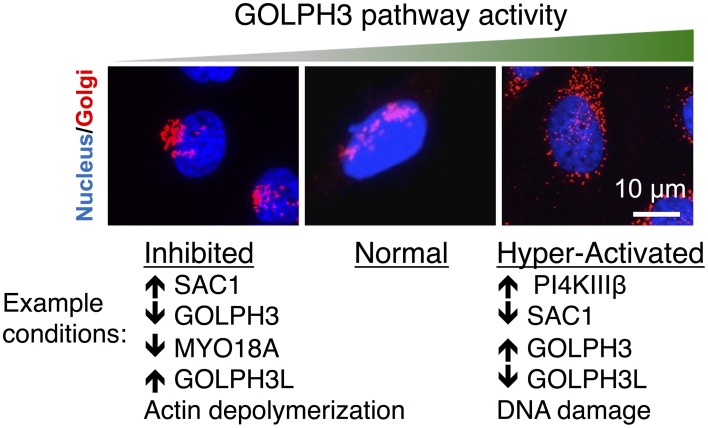
**Perturbations of the GOLPH3 pathway alter Golgi morphology**. Examples of the Golgi (in red, co-stained with the nucleus in blue) are shown under normal conditions and in response to perturbations to interfere with or to increase the activity of the GOLPH3 pathway. Normally the Golgi appears as an extended ribbon partially wrapped around the nucleus (middle). Inhibition of the GOLPH3 pathway, by overexpression of SAC1 (Dippold et al., 2009), siRNA knockdown of GOLPH3 or MYO18A (Dippold et al., 2009; Ng et al., 2013; Farber-Katz et al., 2014), overexpression of GOLPH3L (Ng et al., 2013), or depolymerization of actin (Dippold et al., 2009), results in compaction of the Golgi (left). Activation of the GOLPH3 pathway, by overexpression of PI4KIIIβ (Hausser et al., 2005) or GOLPH3 (Ng et al., 2013), siRNA knockdown of SAC1 (Liu et al., 2008) or GOLPH3L (Ng et al., 2013), or in response to DNA damage (Farber-Katz et al., 2014), results in extension and dispersal of the Golgi (right).

Depletion of PtdIns(4)P, GOLPH3, MYO18A, or F-actin results in both a defect in Golgi-to-plasma membrane trafficking and also a striking change in the morphology of the Golgi (Figure 2). The normal Golgi ribbon that extends partially around the nucleus condenses into a compact ball at one end of the nucleus upon interference with any component of the GOLPH3 pathway. For example, depletion of PtdIns(4)P by overexpression of SAC1, siRNA knockdown of GOLPH3 or MYO18A, or depolymerization of F-actin by latrunculin B, all cause Golgi compaction (Dippold et al., 2009). From this and other data (see Dippold et al., 2009) we infer that normally the GOLPH3 pathway exerts a tensile force upon the Golgi that contributes to stretching the Golgi around the nucleus. However, the fact that the GOLPH3 pathway is conserved across species, even those with dramatically different Golgi morphologies, suggests that imparting this shape is not the primary role of the pathway, but rather is a side-effect of its role in GOLPH3-dependent Golgi-to-plasma membrane trafficking.

While interfering with the GOLPH3 pathway results in Golgi compaction, increasing the activity of the GOLPH3 pathway causes expansion of the Golgi (Figure 2). For example, mild overexpression of GOLPH3 leads to an increasingly extended Golgi ribbon. However, very high levels of overexpression of GOLPH3 result in fragmentation and dispersal of the Golgi throughout the cytoplasm (Ng et al., 2013). Likewise, an increase in PtdIns(4)P at the Golgi by knockdown of SAC1 (Liu et al., 2008) or overexpression of PI4KIIIβ (Hausser et al., 2005) also result in increasing Golgi extension and even dispersal of the Golgi. Moderate increases in GOLPH3 pathway activity lead to Golgi extension and mild increases in anterograde trafficking. However, large increases in GOLPH3 pathway activity result in dramatic Golgi fragmentation and impaired trafficking (Ng et al., 2013; Farber-Katz et al., 2014).

## Regulation of golgi morphology and function via the GOLPH3 pathway

A growing body of evidence demonstrates regulation of Golgi morphology and function via the GOLPH3 pathway (Figure 1). For example, growth factor signaling regulates PtdIns(4)P levels at the Golgi by regulating SAC1 localization. In response to growth factor signaling, activated p38/MAPK phosphorylates SAC1, causing it to translocate from the Golgi to the ER (Blagoveshchenskaya et al., 2008). The resulting reduction of PtdIns(4)P-4-phosphatase activity at the Golgi leads to a rise in Golgi PtdIns(4)P levels, with a consequent increase in Golgi-to-plasma membrane trafficking and extension and dispersal of the Golgi.

The Golgi is also regulated by GOLPH3L, a paralog of GOLPH3 that is expressed in highly secretory cells in vertebrates (Ng et al., 2013). GOLPH3L binds to PtdIns(4)P and localizes to the Golgi similarly to GOLPH3. However, unlike GOLPH3, GOLPH3L does not bind to MYO18A, and thus acts as an endogenous dominant-negative inhibitor of the GOLPH3 pathway. Our evidence suggests that GOLPH3L acts as a throttle in highly secretory cells to prevent overly aggressive anterograde trafficking. Correspondingly, overexpression of GOLPH3L impairs GOLPH3 pathway function, resulting in Golgi compaction and impaired Golgi-to-plasma membrane trafficking. Knockdown of GOLPH3L results in dramatic Golgi dispersal and impaired Golgi-to-plasma membrane trafficking, similar to the effect of dramatic overexpression of GOLPH3.

## Regulation of the golgi by DNA damage via the GOLPH3 pathway

A recent study by our lab identified novel signaling that regulates the Golgi in response to DNA damage (Farber-Katz et al., 2014). We found that DNA damage triggers dramatic fragmentation and dispersal of the Golgi. The mechanism involves activation of the GOLPH3 pathway. In response to DNA damage, the DNA-damage response kinase, DNA-PK, phosphorylates GOLPH3 on a conserved TQ motif. The phosphorylation of GOLPH3, in turn, enhances its physical interaction with MYO18A, increasing GOLPH3 pathway-dependent vesiculation of the Golgi. As a consequence of this increased GOLPH3/MYO18A interaction, the Golgi undergoes dramatic fragmentation and dispersal, resulting in impaired Golgi-to-plasma membrane trafficking. Thus, DNA damage regulates Golgi shape and function by activating the GOLPH3 pathway.

DNA damage has been posited to contribute to the pathophysiology of many human diseases, including cancer (reviewed in Lord and Ashworth, 2012) and neurodegenerative diseases (reviewed in Madabhushi et al., 2014). Whether DNA damage-induced regulation of the Golgi contributes to the pathophysiology of these diseases remains unknown.

## Relationship between golgi morphology and secretory function

While perturbations of the GOLPH3 pathway result in predictable and consistent changes in both Golgi shape and secretory function, it does not follow that there is a simple relationship between morphology and function. In fact, perturbations of pathways other than the GOLPH3 pathway have been demonstrated to alter Golgi morphology without affecting secretory trafficking. For example, depolymerization of microtubules by treatment with nocodazole leads to Golgi fragmentation and dispersal (Thyberg and Moskalewski, 1999), but does not alter secretory trafficking (Hirschberg et al., 1998). Similarly, depletion of Rab29, or inhibition of Rab29 function by overexpression of the inactive mutant Rab29-T21N results in fragmentation of the Golgi (Wang et al., 2014). However, despite the dramatic change in Golgi morphology, no change is observed in trafficking of VSVG to the plasma membrane.

Thus, we conclude that there is not a simple relationship between Golgi morphology and secretory function that holds under all circumstances. Instead, the effect of a perturbation on the Golgi is dictated by the specific mechanism of the perturbation. However, for perturbations of the GOLPH3 pathway the consequences to Golgi morphology and secretory function are predictable.

## Functional consequences of altered golgi

Since changes in Golgi morphology do not directly relate to its trafficking function, it remains unclear whether changes in Golgi morphology always have cellular consequences. However, it seems safe to predict that perturbations of the Golgi that affect Golgi-to-plasma membrane trafficking will have consequences to the cell. The phenotypes associated with perturbation of the GOLPH3 pathway provide strong examples of the importance of Golgi-to-plasma membrane trafficking to overall cell and organismal biology. Notably, GOLPH3 is an oncogene that is frequently amplified and overexpressed in human cancers, and its increased expression predicts poor patient prognosis (Scott et al., 2009 and reviewed in Buschman et al., 2015). Furthermore, other components of the GOLPH3 pathway have been implicated in oncogenesis as well. Increased expression of PI4KIIα protein and PI4KIIIβ mRNA have been detected in cancer cells (Li et al., 2010; Curtis et al., 2012). A recent unbiased computational analysis of data from The Cancer Genome Atlas for breast cancer has identified MYO18A as a cancer driver (Sanchez-Garcia et al., 2014).

PtdIns(4)P and GOLPH3 have been demonstrated to play important roles in regulating cell motility. Knockdown of SAC1, resulting in elevated Golgi PtdIns(4)P levels, leads to increased cell migration (Tokuda et al., 2014). Conversely, knockdown of PI4KIIIβ, resulting in a reduction of Golgi PtdIns(4)P levels, impairs cell migration. These consequences of modulation of PtdIns(4)P levels are mediated by its downstream effector GOLPH3. Likewise, perturbation of GOLPH3 itself results in similar effects on cell migration. Increased GOLPH3 promotes cell migration and invasion, while depletion of GOLPH3 impairs cell migration (Isaji et al., 2014; Tokuda et al., 2014).

Our recent study on GOLPH3-mediated Golgi dispersal/fragmentation upon DNA damage has revealed an interesting role of the pathway in promoting cell survival (Farber-Katz et al., 2014). We have demonstrated that the GOLPH3-dependent Golgi response to DNA damage is functionally important for cell survival after DNA damage. Depletion of GOLPH3, MYO18A, or DNA-PKcs not only prevents dispersal of the Golgi, but also significantly increases apoptosis and reduces cell survival after treatment with DNA-damaging agents. Conversely, overexpression of GOLPH3 confers a survival advantage to cells after DNA damage. Importantly, this survival advantage caused by GOLPH3 overexpression requires both GOLPH3 localization to the Golgi and its phosphorylation by DNA-PK. Altered Golgi-to-plasma membrane trafficking that occurs upon Golgi fragmentation after DNA damage is believed to be important for the enhanced survival benefit provided by this pathway, although the critical cargoes have not been identified as of yet. The DNA damage response is a well-known driver of cancer progression (reviewed in Lord and Ashworth, 2012). In addition, the resilience against DNA damage conferred by overexpression of GOLPH3 has particular relevance to cancer, as most standard cancer therapeutic regimens include DNA-damaging agents. Therefore, our findings raise hope of using GOLPH3 expression levels as a clinical marker to predict responsiveness to DNA-damaging cancer therapies. Furthermore, novel agents to interfere with the GOLPH3 pathway may have therapeutic benefit when used in combination with standard DNA-damaging therapeutic agents.

## Potential role of the GOLPH3 pathway in neurodegenerative disease

Whether the GOLPH3 pathway plays a role in the pathophysiology of neurodegenerative disease has not been studied. However, some of the known pathophysiology of neurodegenerative disease makes it reasonable to speculate that the GOLPH3 pathway may be involved. First, dysregulation of the Golgi is a common feature of a variety of human neurodegenerative diseases, including amyotrophic lateral sclerosis, Parkinson's disease, and Alzheimer's disease. The accompanying articles in this issue of Frontiers in Neuroscience provide detailed reviews of the literature implicating Golgi dysregulation in neurodegenerative disease. We note that the changes in Golgi morphology that are associated with these diseases mimic those seen with perturbations of the GOLPH3 pathway. Moreover, knockout of a Golgi-specific PI-4-kinase, PI4KIIα, expected to interfere with GOLPH3 pathway function, leads to late onset neurodegenerative disease in mice (Simons et al., 2009). These knockout mice develop cerebellar Purkinje cell loss and spinal cord axonal degeneration, which closely resembles hereditary spastic paraplegia. Thus, there is at least reason to speculate a role for the GOLPH3 pathway in neurodegenerative disease.

A second link to the GOLPH3 pathway arises from a substantial body of literature implicating the DNA damage response in neurodegenerative disease. Loss-of-function mutations in various DNA damage repair genes cause a range of neurodegenerative diseases (Madabhushi et al., 2014). For example, the DNA damage activated protein kinases ATM and ATR (paralogs of DNA-PK) are mutated in ataxia-telangectasia and ATR-Seckel syndrome, respectively (McKinnon, 2012). Recent data indicate that DNA breaks occur frequently in neurons (Madabhushi et al., 2015), bolstering the concern that dysregulated DNA damage repair may contribute to disease in neurons.

Our discovery that the Golgi is regulated in response to DNA damage via DNA-PK activation of the GOLPH3 pathway provides a plausible link between the evidence implicating DNA damage and altered Golgi function in neurodegenerative disease.

## Concluding remarks

The appearance of the Golgi, at least in part, is a consequence of the forces that act on it for vesicle trafficking. The GOLPH3 pathway, in particular, demonstrates the relationship between Golgi secretory function and Golgi morphology. As might be expected for a pathway with an important role in Golgi function, the GOLPH3 pathway is a target for regulation by multiple signals. For example, the GOLPH3 pathway is activated in response to DNA damage, with consequences for Golgi shape and trafficking function, as well as cell survival. Since many proteins depend on trafficking from the Golgi for their function, alterations in GOLPH3-dependent Golgi-to-plasma membrane trafficking have pleiotropic effects on cellular function, significantly impacting biology and human pathophysiology.

## Funding

MB is funded by the American Cancer Society by the Lee National Denim Day Postdoctoral Fellowship Award PF-13-367-01-CDD. SF is funded by an Era of Hope Scholar Award from the Breast Cancer Research Program of the Department of Defense W81XWH-10-1-0822 and the Burroughs Wellcome Fund Career Award in the Biomedical Sciences.

### Conflict of interest statement

The authors declare that the research was conducted in the absence of any commercial or financial relationships that could be construed as a potential conflict of interest.
